# Na_9_Bi_5_Os_3_O_24_: A Diamagnetic Oxide Featuring a Pronouncedly Jahn–Teller‐Compressed Octahedral Coordination of Osmium(VI)

**DOI:** 10.1002/anie.202103295

**Published:** 2021-06-17

**Authors:** Gohil S. Thakur, Hans Reuter, Alexey V. Ushakov, Gianpiero Gallo, Jürgen Nuss, Robert E. Dinnebier, Sergey V. Streltsov, Daniel I. Khomskii, Martin Jansen

**Affiliations:** ^1^ Max Planck Institute for Chemical Physics of Solids Nöthnitzerstr. 40 01187 Dresden Germany; ^2^ Faculty of Chemistry and Food Chemistry Technical University 01069 Dresden Germany; ^3^ Institute for Chemistry of New Materials University of Osnabrück Barbarastraße 7 49069 Osnabrück Germany; ^4^ M. N. Mikheev Institute of Metal Physics Ural Branch of Russian Academy of Sciences 620041 Ekaterinburg Russia; ^5^ Max Planck Institute for Solid State Research Heisenbergstr. 1 70569 Stuttgart Germany; ^6^ Ural Federal University 620002 Ekaterinburg Russia; ^7^ II. Physikalisches Institut Universität zu Köln Zülpicher Str. 77 50937 Köln Germany

**Keywords:** hydrothermal synthesis, Jahn–Teller compression, multinary osmate, spin–orbit coupling

## Abstract

The Jahn–Teller (JT) theorem constitutes one of the most fundamental concepts in chemistry. In transition‐element chemistry, the 3d^4^ and 3d^9^ configurations in octahedral complexes are particularly illustrative, where a distortion in local geometry is associated with a reduction of the electronic energy. However, there has been a lasting debate about the fact that the octahedra are found to exclusively elongate. In contrast, for Na_9_Bi_5_Os_3_O_24_, the octahedron around Os^6+^(5d^2^) is heavily compressed, lifting the degeneracy of the *t_2g_* set of 5*d* orbitals such that in the sense of a JT compression a diamagnetic ground state results. This effect is not forced by structural constraints, the structure offers sufficient space for osmium to shift the apical oxygen atoms to a standard distance. The relevance of these findings is far reaching, since they provide new insights in the hierarchy of perturbations defining ground states of open shell electronic systems.

## Graphical abstract

A Jahn–Teller‐compressed OsO_6_ octahedron resulting in an unprecedented large splitting of the *t*_2g_ levels and a diamagnetic ground state has been observed.

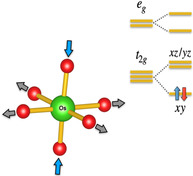

## Introduction

Dealing with multitudes of particles constitutes an inescapable and major challenge in chemistry, even within the framework of the Born‐Oppenheimer approximation. While for molecular species the numbers of atoms involved are finite, they are virtually infinite for extended solids. In coping with the task of structuring and classifying chemical knowledge for purposes of for example, communication, teaching, understanding or planning of chemical research the enormous richness of empirical and computational results need to be cast into comprehensible concepts.^[1]^ Along the most popular of such approaches, one tries to structure observations, and even to derive cause‐effect relationships, by breaking down collective properties into additive increments. Prominent examples are atomic radii (from distances),^[2]^ electronegativity (from shortening of covalent bonds due to polarity),^[3]^ oxidation states (assigning charges by systematic definition),^[4]^ or the reverse, bulk diamagnetic response of a compound (from calculated increments).^[5]^ Such procedures are most reliable for compounds consisting of only two different elements, making it easier to separate the individual contributions of the constituting atoms. However, as a distinct disadvantage of such binaries, the degrees of freedom in evolving different compositions, structures, bonding schemes and properties are substantially limited. With respect to both these issues, the opposite holds true for multinary compositions, and the individual constituting atom types are less constrained in striving for their respective pertinent preferred configurations.

Here we report on an illustrative example of a novel four‐component oxide Na_9_Bi_5_Os_3_O_24_ in which the additional degrees of freedom have enabled a complex composition and a singular crystal structure to form, where the constituents Na, Bi, Os, and O come close to their characteristic structure and bonding properties. As a surprising result, although containing Os^6+^ with a 5*d* ^2^ electron configuration, the compound is diamagnetic and exhibits a substantially compressed octahedron around osmium, which causes an exceptionally strong crystal field splitting of the *t_2g_* levels thus lifting the degeneracy of unequally occupied orbitals according to a Jahn–Teller (JT) effect. This finding is very special in another, more general respect. In known solids the transition metal (TM) ions with partially‐filled *t_2g_* levels always obey the first Hund's rule, that is, electrons are arranged such that the maximum S results. We are not aware of any exception of this rule for *t_2g_* shells in extended solids. In one incident, La_4_Ru_2_O_10_, it was initially suggested that Ru^4+^ has an *S*=0 ground state.^[6]^ This was challenged later, and shown that the nonmagnetic character of this system is due to the formation of singlet Ru dimers (“molecules in solids”).^[7]^ In our case, as we show below, Os^6+^(5*d* ^2^) really has a singlet ground state with *S*=0, violating the first Hund's rule. This particular configuration may form because of a synergetic action of weaker structural matrix effects of the many‐component material and a strong local JT effect, seen for the first time to act on the *t_2g_* levels of an octahedral complex and to induce a compression.

## Results and Discussion

### Synthesis and Chemical Properties

**Figure 1:**
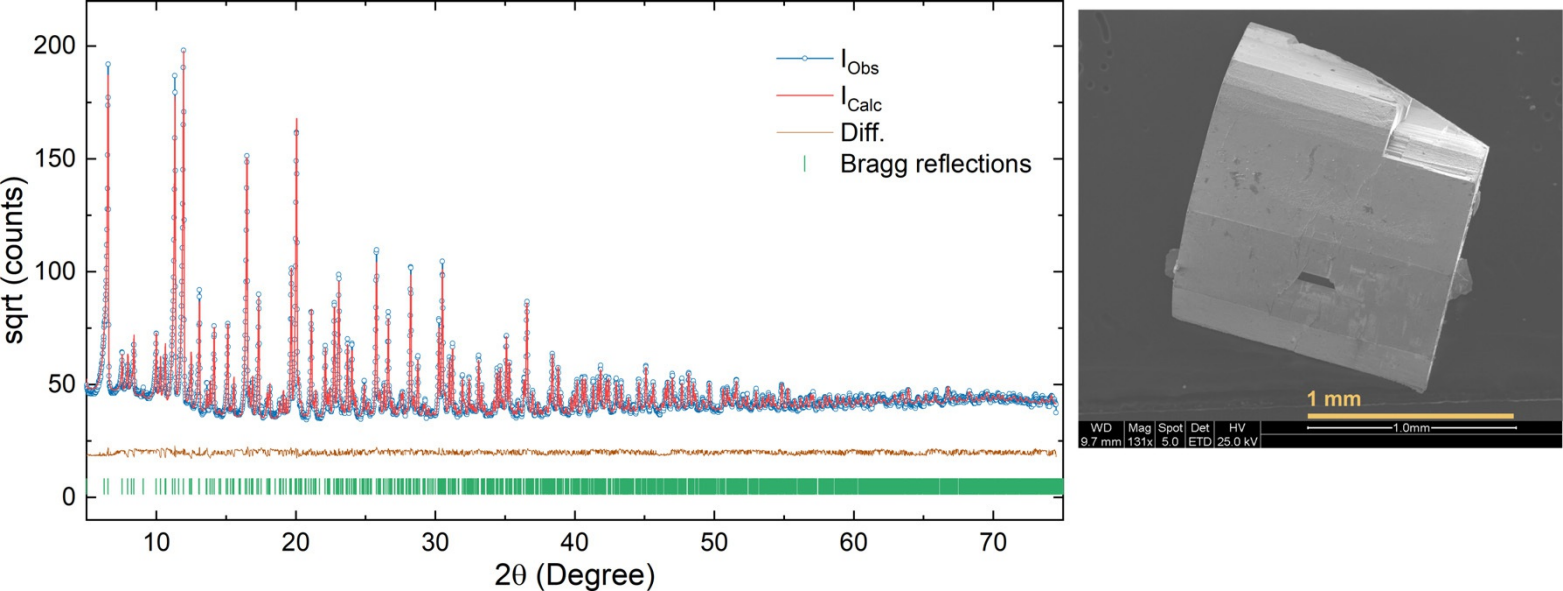
Rietveld fit to the powder X‐ray diffraction pattern of Na_9_Bi_5_Os_3_O_24_. Blue, red and brown lines are the observed, calculated and difference curve, respectively. The green vertical bars represent the allowed Bragg reflections. To the right is the electron microscopic image of a typical crystal.

Na_9_Bi_5_Os_3_O_24_ was obtained by reacting the binary constituent oxides at high oxygen pressure and hydrothermal conditions. Black reflective hexagonal blocks or thick plates were harvested after washing the product with water and rinsing with ethanol (Figure 1). Several crystals from different batches were quantitatively analyzed using SEM‐EDX, which showed an approximate metal ratio of Na/Bi/Os=3.1/1.8/1 (Figure S1 in Supporting Information (SI)). A composition of Na_8.63_Bi_4.81_Os_3.0_O_24.4_ was estimated from ICP‐OES and AAS analysis of the metal concentrations (see Table S1 in SI) basically confirming the composition obtained from EDAX. The compound starts decomposing in a stepwise manner at 600 K (See Figure S2 in SI) but the decomposition is not complete till 1273 K.

The powder X‐ray diffraction pattern was refined using the final atomic parameters from the single crystal structure determination as a starting model. No apparent impurity phase was observed in the powder pattern. Figure 1 shows the result of a Rietveld profile refinement, for more details see, Tables S2 and S3 in SI.

### Crystal Structure

The determination of the crystal structure by X‐ray diffraction did not proceed straight forwardly and required to employ both powder and single crystal techniques in an alternating fashion, for details see experimental section and Tables S4–S7 in SI.

**Figure 2:**
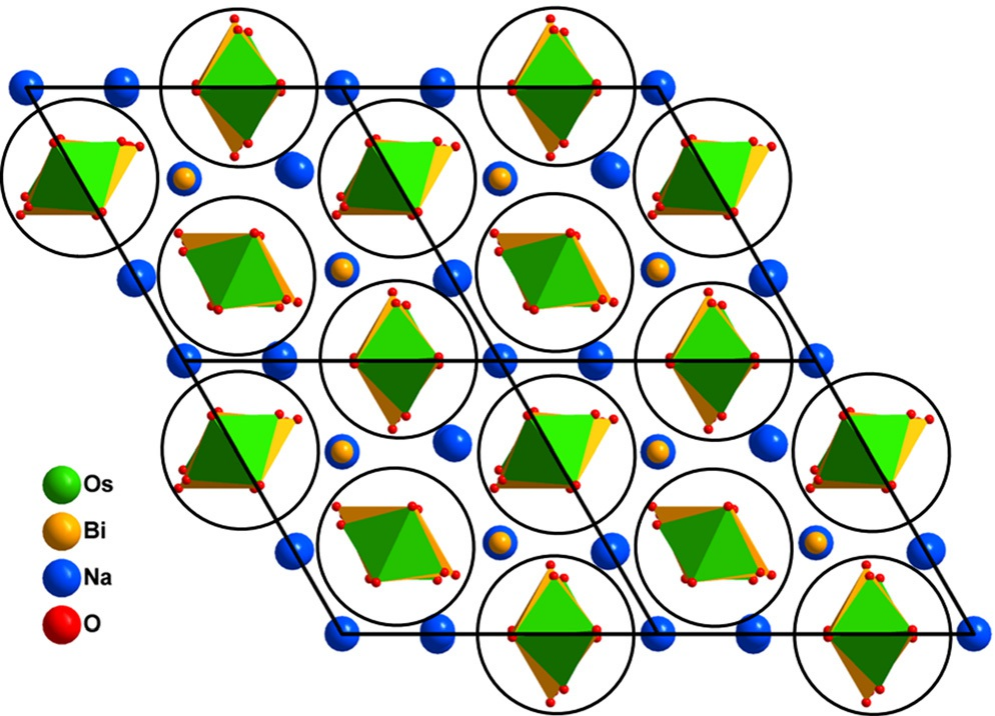
Projection of the crystal structure, view direction [001], strands of alternating OsO_6_ and BiO_6_ octahedra are emphasized by black circles.

**Figure 3:**
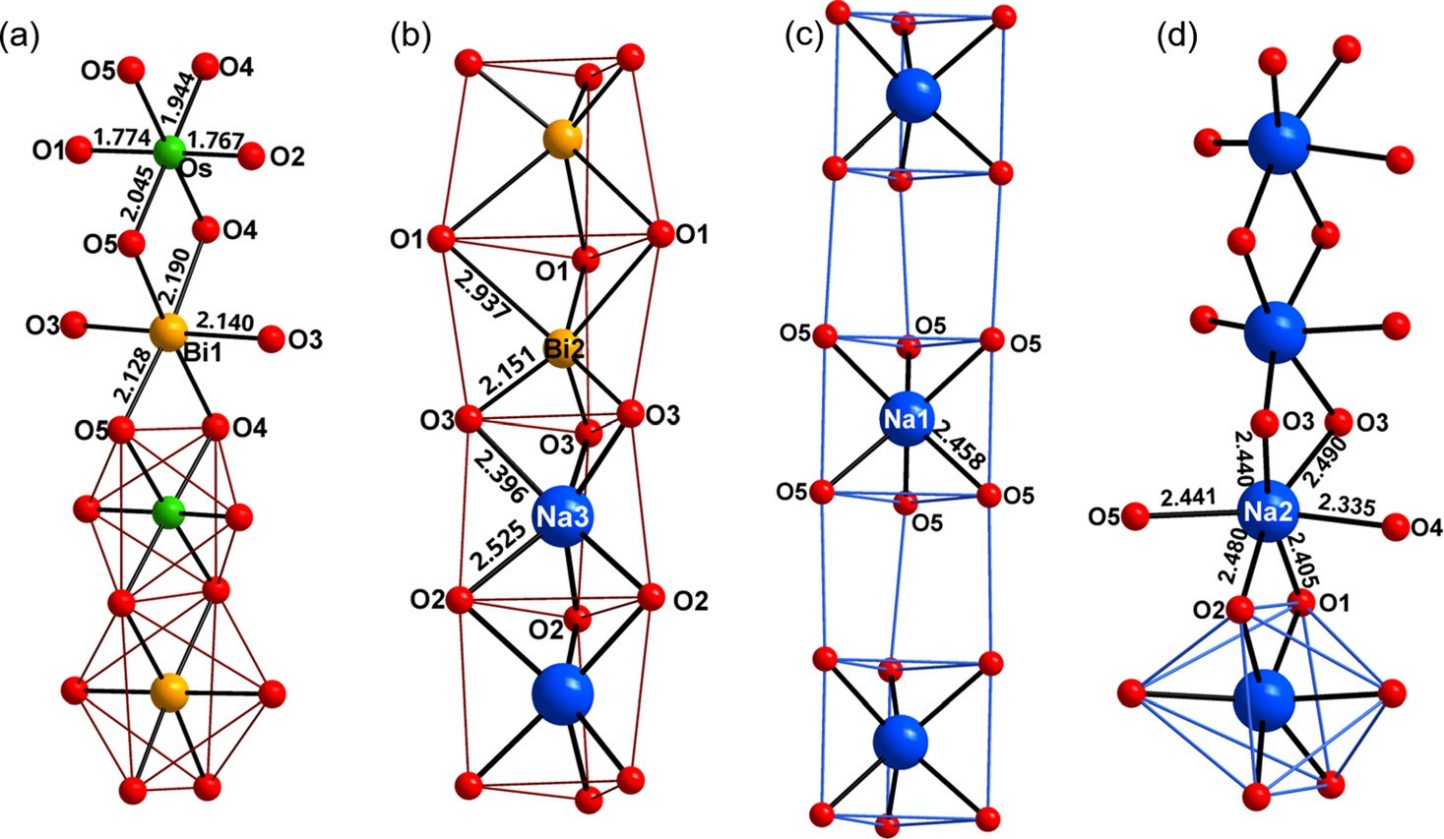
Presentation of the individual quasi‐1D secondary building units, a) chains of edge‐shared alternating Os/Bi octahedra, b) chains of alternating pairs of trigonal prisms of Na3/Bi2, c) chains of alternating empty and filled (Na1)O_6_ trigonal prisms and d) chains of edge shared distorted (Na2)O_6_ octahedra, for details compare text. Bond lengths are in Å.

**Figure 4:**
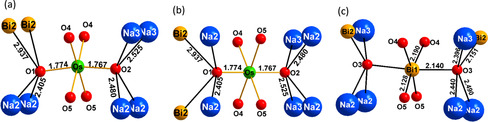
First coordination sphere of osmium(VI), a) view direction perpendicular to, b) within the crystallographic mirror plane, and c) of bismuth(V). For osmium, the apical oxygen atoms are in a different local environment, generating local dipole moments, which completely compensate due to space group symmetry. Bond lengths are in Å.

From the structure and chemical formula it was deduced that osmium is hexavalent and bismuth is mixed‐valent with three out of five (Bi1) being pentavalent and the remaining trivalent (Bi2). Na_9_Bi_5_Os_3_O_24_ exhibits a singular and peculiar crystal structure that cannot be related to any of the prominent aristotypes of typical oxide structure families. Here, for providing a comprehensive description, we resort to the tool of analyzing extended structures in terms of rod packings.[^8^, ^9^] As is emphasized in Figure 2, all secondary building units are quasi‐1D and form chains extending in *c* direction of the hexagonal crystal system. Strands of edge sharing octahedra, centered in an alternating fashion by Os^6+^ and Bi^5+^, are dominating and constitute a precisely hexagonal rod packing. All oxygen atoms in the structure are attached to these chains. Positions and orientations of the latter generate columns of trigonal prisms around the threefold axes of the space group at 1/3 2/3 *z* (1), 2/3 1/3 *z* (2) and 0 0 *z* (3), while a fourth column of oxygen atoms around Na2 is not subject to space group symmetry constraints. The individual rods are shown in Figure 3. The respective local coordinations for the chain of edge sharing octahedra show a typical pattern for Bi^5+^, however, an extremely compressed octahedron around Os^6+^, which is a conspicuous structural feature for this ion. Interestingly, the trigonal prisms of columns (1) and (2) are each occupied by trivalent Bi2 and Na3 in a pairwise alternating fashion, instead of simply alternating, which would be favorable for electrostatic reasons. The coordination polyhedra observed for these atoms are basically as expected. Bismuth(III) forms a trigonal pyramid with three short bonds, and three distinctly longer ones, in accord with the “active lone‐pair” scenario.[^10^, ^11^] Na3 as well shows common distances towards oxygen on average, however, is seemingly unforced in an off‐center position. The trigonal strands around the origin of the unit cell comprise alternatingly filled, (Na1)O_6_, and empty regular trigonal prisms. Within the remaining chain, Na2 is coordinated by six nearest oxygen atoms, forming a substantially distorted octahedron. These polyhedra are connected to chains oriented along [001] via common edges O1‐O2 and O3‐O3, respectively. The rods are linked to form a three‐dimensional framework, basically via the apical oxygen atoms of the Bi^5+^ and Os^6+^ octahedra. Now, the key role played by the distorted trigonal prisms around trivalent Bi2 and Na3 in controlling the bond length to the apical oxygen atoms is becoming evident: The particular sequence of Bi2 and Na3 is generating a breathing mode of the oxygen atoms reflected by small triangles between Bi2 and Na3 atoms and wider ones between each two subsequent Bi2 and Na3, respectively. The oxygen atoms constituting the smaller triangles are giving space to Bi^5+^ to adopt a virtually regular octahedron, while the wider triangles enable Os^6+^ to develop the strikingly short bonds to the apical oxygen atoms, that is, to form a substantially compressed octahedron. Interestingly, this contraction is not forced by any matrix effect of the crystal structure. From Figure 4 one can see that there is sufficient freedom within the linkages Os‐O1‐Bi2 and Os‐O2‐Na3 to shift the respective oxygen atoms away from osmium. Even in the extreme, when moving O2 into the barycenter of the quadrilateral formed by Na2(2×) and Na3(2×), the Na‐O separations are still within the common limit, see Figure S3. Thus, from a crystal chemistry point of view, there must be local electronic effects that are driving osmium into that peculiar configuration observed experimentally.

As one of the remarkable characteristics of the crystal structure found, cations as different as Bi^3+^ and Na^+^ (Bi2 and Na3) are hosted in virtually the same kind of trigonal prisms, see Figure 3 b. Since this might cause some anti‐site disorder between these two positions, we refined the respective site occupation factors. Indeed, slight indications were obtained for a low degree of anti‐site disorder, exclusively for these two positions. The site occupation factor (SOF) of 96.53 and 3.47 (%) obtained is at the limits of experimental reliability, since SOF and temperature parameters are commonly strongly correlated in the refinements. This finding does not question the characteristics of the crystal structure, in particular not the short bonds between osmium and the apical oxygen atoms, since the anti‐site disorder only inverts the two different surroundings of the apical oxygen atoms. At screening crystals from different batches we became aware that this disorder may vary from one synthesis batch to the other, depending on the temperature schedules applied. At the extreme, a SOF ratio of 39.58 % to 60.42 % was encountered. Data of the split atom refinements are included in SI as Tables S8—S12.

### Physical Properties

**Figure 5:**
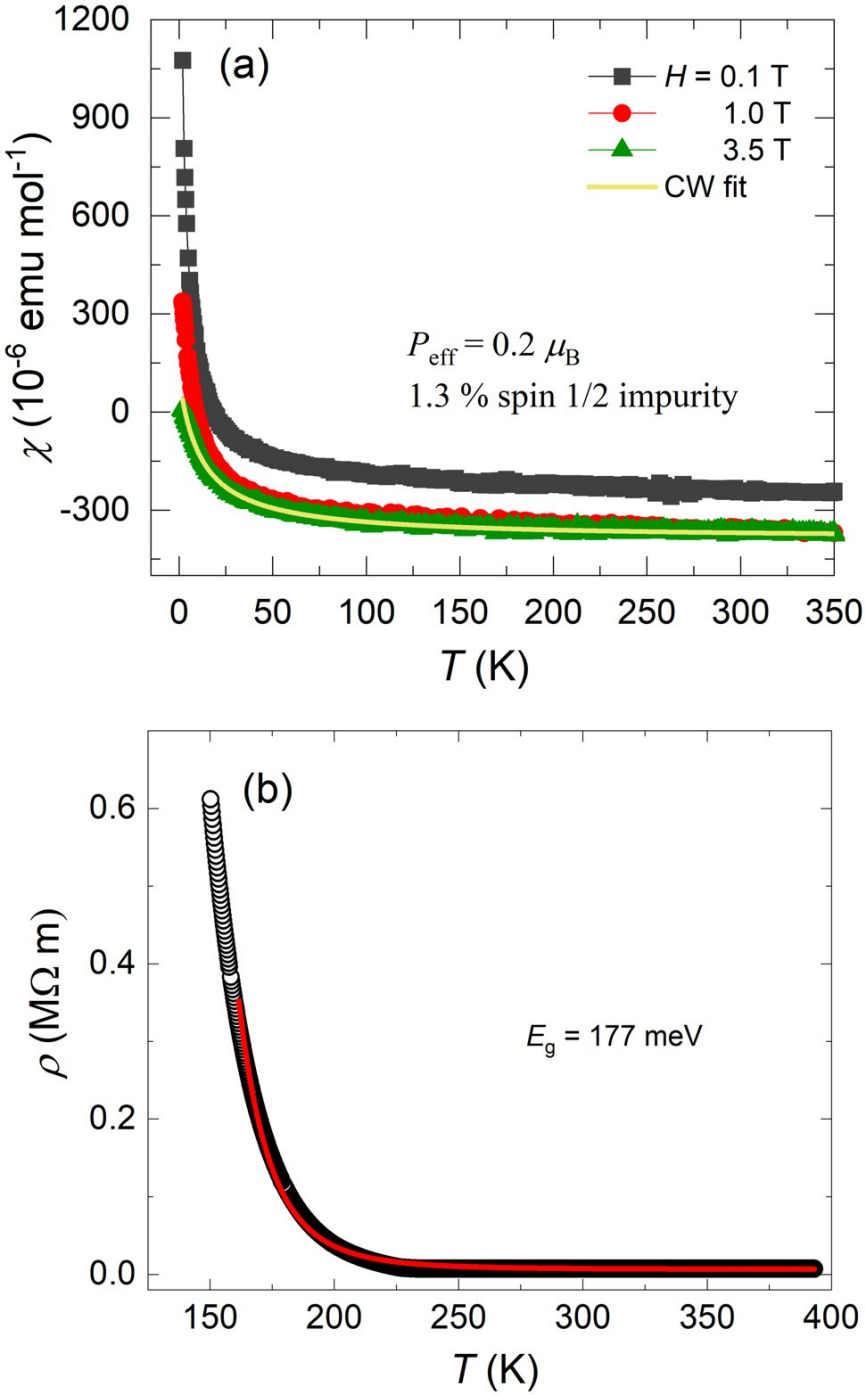
Temperature dependent magnetic susceptibility and its Curie fit (*χ*=*C*/*T*) (a) and Arrhenius fit of electrical resistivity (b) of Na_9_Bi_5_Os_3_O_24_ single crystal(s).

Figure 5 a shows the magnetic susceptibility measured in the temperature range of 2–350 K at different applied magnetic fields (0.1, 1.0, 3.5 T), clearly indicating the diamagnetic nature of the sample. Minor Curie tailing observed at low temperatures corresponds to an upper estimate of a magnetic moment *P*_eff_=0.2 μ_B_ mol^−1^ corresponding to 1.3 % of a spin 1/2 impurity (from a Curie fit of the susceptibility in the entire temperature range). Electrical resistivity data (Figure 5 b) shows that the compound is semiconducting with a band gap of 177 meV.

### Computational Results and Theoretical Analyses

**Figure 6:**
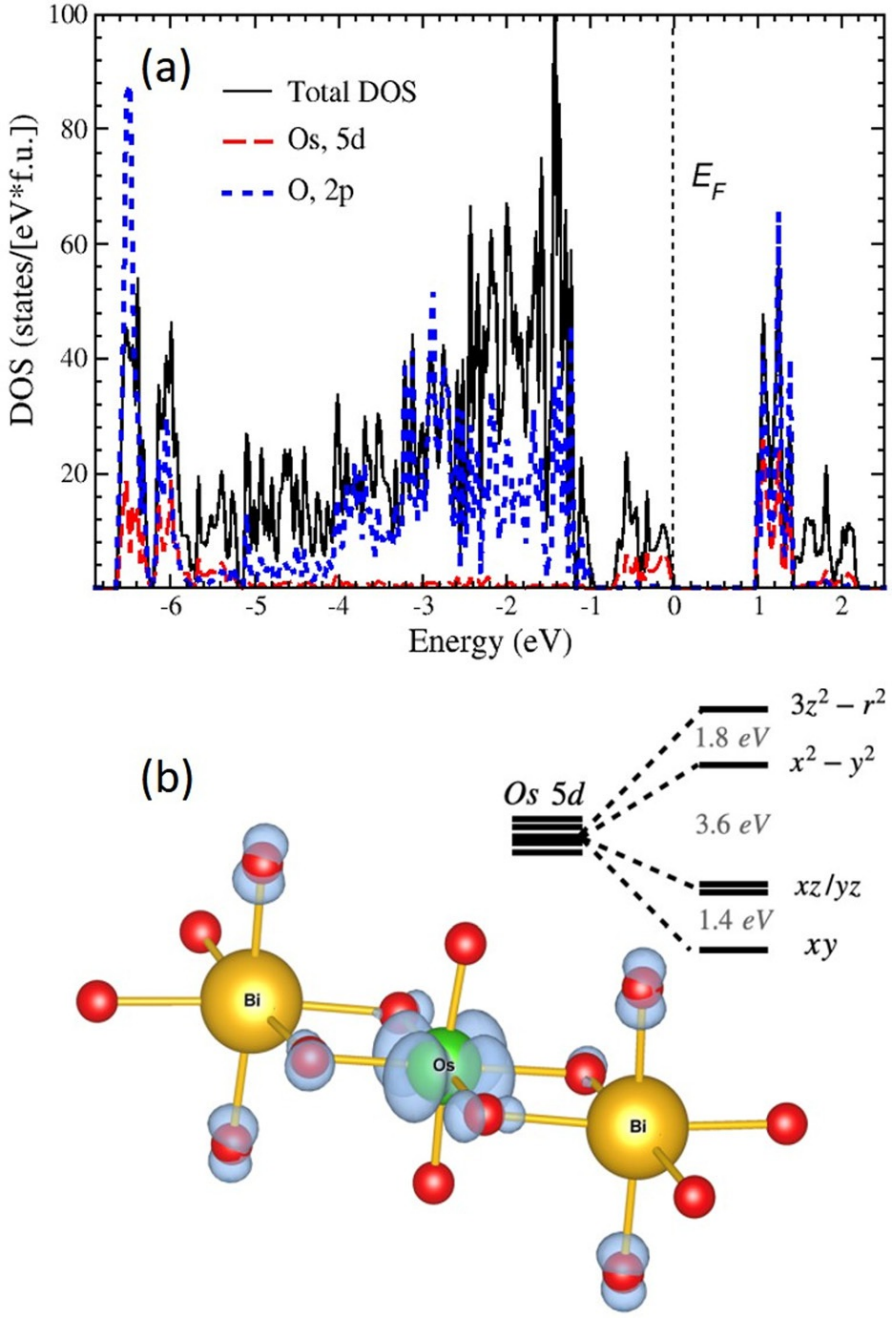
Total and partial density of states plots (a) and charge density (blue) corresponding to a single occupied band (just below the Fermi level) in the GGA+U calculation (b). This band has predominantly Os d states character. Osmium (oxygen) atoms are shown by green (red) balls. Inset of (b) shows Os 5d level scheme; note, there is also a small splitting of ≈10 meV in the *xz*/*yz* manifold.

In order to uncover the physical origin of the low‐spin state stabilization in Na_9_Bi_5_Os_3_O_24_, we calculated the crystal‐field splitting of the Os *t_2g_* sub‐shell in the DFT approach (see Figure 6) and found that it amounts to 1.4 eV, with the singlet *xy* orbital lying below the doublet *xz*, *yz* (in the local coordinate system with axes directed from Os towards oxygen atoms, *z* direction to the apical ligands). This separation virtually corresponds by magnitude to the octahedral *t_2g_*‐*e_g_* splitting (*10Dq*) in conventional *3d* transition metal oxides. Considering Hund's energy for *5d* ions of ≈0.5 eV,^[12]^ it is well understandable that the lowest *xy* orbital is filled by two electrons of Os^6+^, resulting in a singlet ground state, *S*=*0*. Thus, it is clearly the strong crystal‐field (ratio of short and long Os−O bonds is ≈0.89), which leads to the non‐magnetic state in the investigated material. It is worthwhile mentioning that, based on various experimental evidences, there have been claims of a low‐spin state realized in *t_2g_* configurations.^[6]^ However, respective theoretical and experimental studies did not confirm such a picture, see[^7^, ^12^] and references therein. To the best of our knowledge Na_9_Bi_5_Os_3_O_24_ is thus the only oxide, where such a situation affecting a set of *t_2g_* levels is actually realized.

For checking the role of the degree of filling of the 5*d* derived states, we performed crystal structure optimizations with an artificially reduced number of electrons in the system of six per formula unit (compensated by a corresponding surrounding charge) or by substituting W^6+^ (5*d* ^*0*^) for Os^6+^ (volume, unit cell shape, atomic positions were allowed to relax). The results are qualitatively similar and rather illustrative. In the first case, of reduced number of electrons, the distance from the plane formed by equatorial oxygen atoms to apical ligands increases (1.87 and 1.97 Å), while the in‐plane M−O bond lengths decrease (1.87 Å(2×) and 1.89 Å(2×)), for positional parameters see Table S13 in SI. When giving the 5*d* ^2^ electrons back to the system (or reinstating Os for W), the structure returns to the original experimental configuration upon computational relaxation. These observations clearly demonstrate that the compression of the OsO_6_ octahedron is basically caused by electronic effects, that is, due to a JT effect lifting the *t_2g_* orbital degeneracy.

The following consideration can be proposed to explain how the anti‐Hund low‐spin state, which normally corresponds to an exited state, can be stabilized in Na_9_Bi_5_Os_3_O_24_. It is imperative to have two electrons on a JT active orbital and lattice connectivity allowing for a strong distortion. In a normal situation for a single *d* electron or hole the decrease in one‐electron energy due to JT effect is −*gδ* (where, *δ* is distortion and *g* is the coupling constant); it competes with the elastic energy ≈*Bδ* ^2^/2, and this would result in JT distortions *δ*=*g*/*B* and a JT energy gain of *E*_JT_=−*g* ^2^/2 *B*.^[13]^ For the low‐spin state of d^2^ configuration we of course lose Hund's coupling energy, but it can be readily seen that in this case the JT distortion is much stronger, *δ*=2 *g*/*B*, and the energy gain is 4 times larger, *E*_JT_=−2 *g* ^2^/*B* (the gain in the one‐electron energy is in general −*gδn*, where n is the number of electrons at the lower orbital).

It appears advisable to compare the situation in Na_9_Bi_5_Os_3_O_24_ with other functional oxides and coordination compounds containing Os^6+^. In principle, open shell 5*d* systems can arrive at a nonmagnetic state due to strong spin‐orbit interaction even for a cubic crystal field. This has been encountered for 5*d* ^4^ ions like Ir^5+^.[^14^, ^15^] Also for Os^6+^ (5*d* ^2^) a nonmagnetic ground state owing to a combined action of spin‐orbit coupling and cubic crystal field (10 *Dq*) has been reported.[^16^, ^17^, ^18^] The character of this state, however, is distinctly different from what we have in Na_9_Bi_5_Os_3_O_24_: it is not a singlet state with *S*=*0*, but a non‐Kramers *e_g_* doublet. Such an electronic state gives rise to low‐lying magnetic states (for double perovskites like Ba_2_MOsO_6_, M=Ca, Mg, Zn the singlet‐triplet gap is estimated as ≈25 meV).^[16]^ This would give strong and temperature‐dependent van Vleck paramagnetism. In our case, however, there is a singlet state of Os^6+^ with *S*=*0*, with the gap to the lowest‐lying magnetic state (Δ_CF_−*J*_Hund_)≈1 eV. Spin‐orbit coupling plays practically no role in its stabilization. DFT+U+SOC calculations fully support this conclusion. The orbital moment was found to be extremely small, ≈10^−3^ μ_B_. For Na_2_OsO_4_ a comparable compression of the (OsO_6_) octahedra was reported, however, the magnetic ground state is not fully diamagnetic, and the findings have not been analysed in terms of a JT effect.^[19]^ Worth mentioning, there is the quite extended family of coordination compounds [(OsO_2_)L_4_] which seemingly constitute another analogy. Here, short Os−O bonds and diamagnetic behavior are understood in terms of local bonding, in particular enforced by (p‐d)π bonding.^[20]^ In the context of the findings presented here, these examples are of less evidential value for proving a JT effect since the very different sets of two apical and four axial ligands alone are providing sufficient reason for the distinct spread of the Os‐O and Os‐L bond lengths found, and are thus not comparable with the case encountered with the title compound, where the distortion and diamagnetic ground state do not result from such external factors of influence.

## Conclusion

We report on new Na_9_Bi_5_Os_3_O_24_ which features a unique crystal structure and an unparalleled electronic ground state: a strongly compressed octahedron of the oxygen atoms coordinating osmium(VI) is giving rise to a nonmagnetic configuration by hosting the 5*d* ^2^ electrons paired in the *d_xy_* derived state. The splitting of the *t_2g_* levels amounts to 1.4 eV, substantially surpassing the Hund's coupling energy for *5d* ions of ≈0.5 eV. This pronounced JT distortion is not enforced by structural matrix effects, the structure would offer sufficient space to enable a virtually regular octahedron without causing strain. Apparently, the energy landscape of configurations for 5*d* ^2^ systems, like Os^6+^, in approximately octahedral coordination by oxygen is rather flat and, depending on the balance of local electronic factors of influence vs. structural constraints, diverse ground states may develop. Against this background it appears rewarding to inspect these kind of competing impacts, structural frustration vs. strive for an as low as possible local electronic state, on the basis of the structure type presented, which offers plenty of options for modifying the relevant conditions by aliovalent or isovalent substitution of any of the cations present. Such computational studies, at best followed by experimental validation, would have the potential to provide valuable insights in the hierarchy of perturbations defining the ground states of open shell transition element compounds.

## Conflict of interest

The authors declare no conflict of interest.

## Supporting information

As a service to our authors and readers, this journal provides supporting information supplied by the authors. Such materials are peer reviewed and may be re‐organized for online delivery, but are not copy‐edited or typeset. Technical support issues arising from supporting information (other than missing files) should be addressed to the authors.

Supplementary
