# Supplementary material for: Na9Bi5Os3O24: A Diamagnetic Oxide Featuring a Pronouncedly Jahn–Teller‐Compressed Octahedral Coordination of Osmium(VI)
Source: Angew Chem Int Ed Engl. 2021 Jun 17;60(30):16500–5. doi: 10.1002/anie.202103295 (PMC8361922; doi:10.1002/anie.202103295)
Supplement: Supplementary file 1 — Supplementary [file ANIE-60-16500-s001.pdf]

Angewandte Chemie
Eine Zeitschrift der Gesellschaft Deutscher Chemiker

Supporting Information

$Na_9Bi_5Os_3O_{24}$: A Diamagnetic Oxide Featuring a Pronouncedly Jahn–Teller-Compressed Octahedral Coordination of Osmium(VI)

*Gohil S. Thakur, Hans Reuter, Alexey V. Ushakov, Gianpiero Gallo, Jürgen Nuss, Robert E. Dinnebier, Sergey V. Streltsov, Daniel I. Khomskii, and Martin Jansen**

anie_202103295_sm_miscellaneous_information.pdf

Table of Contents

1. Experimental section

1.1 Synthesis……..…………….………………………………………….……….…….….….…….……..…3

1.2 Chemical characterization………..………………………………..…….….….….….….…….……3

1.3 Crystal structure determination

1.4 Physical property measurement…..……………………………..….….…….….……….……....4

1.5 Computational details………..……………………………………...…….….….…….…….……3

2. Tables

Table S1. Wet chemical (ICP-OES/ AAS) analysis. Mole fraction is normalized to Os..............……………**4**

Table S2. Atomic coordinates for $Na_9Bi_5Os_3O_{24}$ obtained by Rietveld refinement of room temperature powder data……………………………………………………………………………………………………**4**

Table S3. Selected bond distances obtained for $Na_9Bi_5Os_3O_{24}$ obtained by Rietveld refinement………………**4**

Table S4. Crystal and structure refinement data for $Na_9Bi_5Os_3O_{24}$ from SCXRD……………………………….**5**

Table S5. Atomic coordinates ($\times$ 10^4) and equivalent isotropic displacement parameters ($Å^2$ $\times$ 10^3) for $Na_9Bi_5Os_3O_{24}$ obtained from single crystal structure refinement. U(eq) is defined as one third of the trace of the orthogonalized Uij tensor……………………………………………………..**5**

Table S6. Selected bond distances for $Na_9Bi_5Os_3O_{24}$……………………………………………………………….**6**

Table S7. Anisotropic displacement parameters ($Å^2$ x 10^3) for $Na_9Bi_5Os_3O_{24}$, ordered structure model. The anisotropic displacement factor exponent takes the form: $-2\pi^2[h^2a^{*2}U_{11} + ... + 2hka^*b^*U_{12}]$………………**6**

Table S8. Crystal data and structure refinement for $Na_9Bi^{V}_3Bi^{III}_2Os_3O_{24}$ assuming disorder……………………**6**

Table S9. Atomic coordinates (x 10^4) and equivalent isotropic displacement parameters (A^2 x 10^3) for crystal 1 and 2 of $Na_9Bi_5Os_3O_{24}$ obtained after refinement (top rows) and with anti-site disorder (bottom rows). U(eq) is defined as one third of the trace of the orthogonalized Uij tensor………………………**7**

Table S10. Summary of bond lengths [Å] obtained from single crystal structure refinement using different protocols of refinement for all crystal samples……………………………………………………………**8**

Table S11. Anisotropic displacement parameters ($Å^2$ $\times$ 10^3) for $Na_9Bi_5Os_3O_{24}$ assuming anti-site disorder (crystal 1). The anisotropic displacement factor exponent takes the form: $-2\pi^2[h^2a^{*2} U_{11} + ... + 2hka^*b^*U_{12}]$………………………………………………………………………………………**8**

Table S12. Anisotropic displacement parameters ($Å^2$ $\times$ 10^3) for $Na_9Bi_5Os_3O_{24}$ assuming inversion and disorder (crystal 2). The anisotropic displacement factor exponent takes the form: $-2\pi^2[h^2a^{*2} U_{11} + ... + 2hka^*b^*U_{12}]$ …………………………………………………………………………………..**9**

Table S13. DFT+U optimized crystal structure (unit cell volume and shape, atomic positions were allowed to relax, number of electrons decreased by 2 per each Os (compensated by external charges)……………………………………………………………………………………………**9**

3. Figures

Figure S1: Elemental analysis of a typical crystal of $Na_9Bi_5Os_3O_{24}$ using SEM-EDX. Inset shows table

of atomic ratios at different regions......10
Figure S2. Thermal decomposition profile of $Na_9Bi_5Os_3O_{24}$......10
Figure S3. Demonstrating lack of structural frustration with respect to short apical Os-O2 bond. The distances are in Å......11

4. References......11

1. Experimental section

1.1 Synthesis: Intimately mixed powders of Bi_2O_3 (0.0777 g, 0.5 mol, Alfa Aesar 99.9 %) OsO_2 (0.1482, 2 mol, Alfa Aesar 99.5%) and Na_2O_2 (0.078 g, 3 mol) to which 0.5 ml of water was added (1 ml of 5 M NaOH solution can be used instead of Na_2O_2), were heated under high oxygen pressure and at high temperature. The solid starting materials were ground in an Ar filled dry glove box and transferred into a gold finger welded at one end. Water was added dropwise outside the glove box immediately before crimping the tube from the top end and placing it in a steel autoclave. The autoclave was tightly sealed after approximately 11.2 ml of liquid oxygen was condensed in it. This amount of oxygen would generate a pressure of around 350 MPa at 773 K. The reaction was carried out for 4 days in a vertical furnace before cooling it naturally. The product was vacuum filtered inside a fume hood and rinsed with distilled water first and subsequently with ethanol. Black reflective hexagonal blocks or thick plates were obtained which were further cleaned and separated by ultra-sonication under ethanol. Crystals of maximum dimensions of up to 1×1×1 mm^3 could be obtained. A white powdery layer on the crystals appears after a few weeks of exposure to moist air, while the powder diffraction pattern did not seem to change. The product was stored in a glove box.

Caution: Under the given conditions, reaction involving osmium generates highly toxic OsO_4, hence, care must be taken during the release of pressure inside the fume hood. Before fetching the product, the opened autoclave was left for several hours inside the fume hood to allow OsO_4 to escape completely. Use of proper PPE (eye protection, respirator as well as latex gloves) is necessary.

1.2 Chemical characterization: Semi-quantitative analysis was performed by scanning electron microscopy-energy dispersive analysis by X-rays (SEM-EDAX). Many crystals from different batches were examined and the data averaged to obtain the metal composition, see Figure S1. Wet chemical analyses of the metal contents were performed by Mikroanalytisches Labor Pascher, An der Pulvermühle 1, D-53424 Remagen, Germany (microwave pressure digestion with HNO_3/HF/HCl; Bi and Os determined by ICP-OES, Na by AAS). Two different runs on the same batch of sample were averaged to obtain the atomic fraction. The results are presented Table S1 in SI. Thermogravimetric (TGA) analysis of the collected single crystals was carried out on a Netzsch STA 449 C analyzer. About 30 mg of the sample was placed in a corundum crucible, which was heated and subsequently cooled at a rate of 5 K min^{-1} in the range of 300–1273 K under dynamic argon flow, see Figure S2.

1.3 Crystal structure determination: The determination of the crystal structure by X-ray diffraction did not proceed straightforwardly and required to employ both powder and single crystal techniques in an alternating fashion.

X-Ray Powder Diffraction (XRPD) measurements were performed using a Stoe Transmission Powder Diffraction System (STADI-P, STOE & CIE, Ge(111) Johansson-type monochromator, $AgK\alpha_1$ radiation (λ = 0.55941 Å)) that was equipped with an array of three linear position-sensitive MYTHEN 1K detectors from Dectris Ltd. of approximately 18° 2θ opening angle each. The finely powdered sample of $Na_9Bi_5Os_3O_{24}$ was placed in a glass capillary of 0.3 mm (Hilgenberg glass No. 14) and spun during measurement for improving particle statistics. The measurement in the range from 1.0 – 111.0° 2θ with a step width of 0.015° 2θ took 3 hrs (Figure 1 in the main manuscript). For indexing of the powder pattern of $Na_9Bi_5Os_3O_{24}$ at T = 298 K, the program TOPAS version 6 (Bruker-AXS, 2018)[1] was used, leading to a hexagonal unit cell with parameters of a = 9.8264(1) and c = 12.8573(2) Å (V = 1075.16(3) $Å^3$). The most probable space groups were determined as P31c (159), $P\bar{3}1c$ (163), $P6_3mc$ (186), $P\bar{6}2c$ (190), and $P\bar{6}_3/mc$ (194) from the observed extinction rules, out of these $P\bar{6}2c$ was confirmed after structure determination. Structure determination of $Na_9Bi_5Os_3O_{24}$ was performed in all possible space groups by the method of Charge Flipping[2], supported by the inclusion of the tangent formula[3] as implemented in TOPAS.[1] The positions of the heavier atoms (Bi, Os) and some candidate for the sodium atoms were found for several space groups but with a clear preference for $P\bar{6}2c$. The space group assumed and the heavy atom structure were confirmed using single crystal X-ray diffraction data, which also enabled to identify the missing light atoms. These final results comply well with the PXRD, as was validated by Rietveld refinement using the TOPAS program. An overall isotropic temperature factor was refined. The profiles related to the final Rietveld refinement are shown in Figure 1 in the main manuscript. The weighted profile R-factor is 3.55 %, the Bragg R-factor is 1.91 %, with a goodness of fit of 2.26. The atomic coordinates are given in Table S2 and a selection of intramolecular distances and angles is given in Table S3.

Crystals suitable for single-crystal X-ray diffraction were selected under highly viscous oil, and mounted with grease on a loop made of Kapton foil (Micromounts™, MiTeGen, and Ithaca, NY). Diffraction data were collected at 298 K with a SMART APEXII CCD X-ray diffractometer (Bruker AXS, Karlsruhe, Germany), using graphite-monochromated Mo-Kα radiation. Reflection intensities were integrated with the SAINT subprogram in the Bruker Suite software,[4] a multi-scan absorption correction was applied using SADABS,[5] and the structure was refined by full-matrix least-square fitting with the SHELXTL software package.[6,7] According to the systematic reflection condition $hh\overline{2h}l$ only present for l = 2n, trigonal space groups P31c (159), P$\bar{3}$1c (163), and hexagonal space groups $P6_3mc$ (186), P$\bar{6}$2c (190) and $P6_3/mmc$ (194) have been checked for structure solution, however, any structure solution method of the SHELXS package failed. The heavy-element positions from powder solutions in the non-centrosymmetric space group P$\bar{6}$2c proved to be a suitable starting model for resolving the complete structure via Difference Fourier analyses. Refinement with anisotropic thermal parameters for all atoms without applying any constraints converged easily. Crystal data and data collection details, positional and isotropic thermal parameters, bond distances and anisotropic thermal parameters respectively, are given in Tables S4-S7. The crystallographic data have been deposited at ICSD under CSD-No. 2063496.

1.4 Physical property measurement: Susceptibility of loose single crystals was measured in applied magnetic fields μ_0H = 0.1, 1.0 and 3.5 T and in the temperature range between 2 and 350 K in a MPMS-XL7 magnetometer (Quantum Design). Temperature dependent resistivity in the temperature range, T = 150–400 K was measured on a block-like single crystal of dimension approximate dimensions 1 × 0.8 × 0.7 mm^3 using a two-probe method in a PPMS instrument (Quantum Design).

1.5 Computational methods: We used the Vienna Ab-initio Simulation Package (VASP) and the generalized gradient approximation for DFT calculations.[8] The on-site Coulomb repulsion U and Hund's intra-atomic exchange J_H parameters were chosen to be 1.1 and 0.5 eV, so that $U-J_H$ = 0.6 eV.[9] A 5×5×5 mesh in k-space was used. For simplicity, we assumed ferromagnetic order (to avoid effects of magnetostriction). Crystal-field splitting parameters were obtained from non-magnetic DFT calculations (computing barycenters of corresponding bands) using Linearized Muffin-Tin Orbitals method.[10]

2. Tables

Table S1. Wet chemical (ICP-OES/ AAS) analysis. Mole fraction is normalized to Os.

Atom	Mass % (1 run)	Mass % (2 run)	Average	Mole fraction	Normalized
Bi	46.5	46.4	46.45	0.2223	4.8
Na	9.34	8.98	9.16	0.3984	8.6
Os	26.5	26.2	26.35	0.1385	3.0
O (remaining)	17.66	18.42	18.04	1.128	24.4

Table S2. Atomic coordinates for $Na_9Bi_5Os_3O_{24}$ obtained by Rietveld refinement of room temperature powder data.

Atom	Wyck.	*x*	*y*	*z*	U [Å^2]
Os1	6h	0.66806	-0.0022	1/4	0
Bi1	6g	0.67116	0	1/2	0
Bi2	4f	1/3	2/3	0.606	0
Na1	2b	0	0	1/4	0
Na2	12i	0.29206	0.98487	0.37917	0
Na3	4f	1/3	2/3	0.39554	0
O1	6h	0.80232	0.1869	1/4	0
O2	6h	0.56344	0.79511	1/4	0
O3	12i	0.52977	0.75106	0.49004	0
O4	12i	0.52719	0.00702	0.37432	0
O5	12i	0.78861	0.99129	0.38295	0

Table S3. Selected bond distances obtained for $Na_9Bi_5Os_3O_{24}$ obtained by Rietveld refinement.

Atom pairs	*Distance* (Å)	Atom pairs	*Distance* (Å)
Os1-O1 (×1)	1.656	Na1-O5 (×6)	2.658
Os1-O2 (×1)	1.725		
Os1-O5 (×2)	2.099	Na2-O4 (×1)	2.211
Os1-O4 (×2)	2.146	Na2-O3 (×1)	2.337
		Na2-O5 (×1)	2.453
Bi1-O5 (×2)	1.925	Na2-O1 (×1)	2.461
Bi1-O3 (×2)	2.129	Na2-O2 (×1)	2.490
Bi1-O4 (×2)	2.171	Na2-O3 (×1)	2.688
Bi2-O3 (×3)	2.243	Na3-O3 (×3)	2.071
Bi2-O4 (×3)	2.798	Na3-O2 (×3)	2.711

Table S4. Crystal and structure refinement data for $Na_9Bi_5Os_3O_{24}$ from SCXRD.

Empirical formula	$Na_9Bi_5Os_3O_{24}$
Formula weight [g/mol]	2206.41
T [K]	296(2)
Crystal system, space group	Hexagonal, $P\bar{6}2c$
a [Å]	9.8115(3)
c [Å]	12.8457(5)
V [Å^3]	1070.93(8)
Z, d_{calc} [g/cm^3]	2, 6.842
μ(Mo$K\alpha$) [mm^{-1}]	58.944
F(000)	1868
$2\Theta_{max}$	70°
Reflections collected	75234
Reflections unique, R_{int}	1637, 0.0877
Data / restraints / parameters	1637 / 0 / 70
Goodness-of-fit on F^2	1.040
R_1, wR2 [I > 2s(I)]	0.0208, 0.0529
R_1, wR2 [all data]	0.0268, 0.0567
Absolute structure parameter	-0.038(6)
Extinction coefficient	0.00102(6)
±Δ [eÅ^{-3}]	1.728/-3.396

Table S5. Atomic coordinates (× 10^4) and equivalent isotropic displacement parameters ($Å^2$ × 10^3) for $Na_9Bi_5Os_3O_{24}$ obtained from single crystal structure refinement. U(eq) is defined as one third of the trace of the orthogonalized Uij tensor.

**Atom**	**site**	***x***	***y***	***z***	**U_{eq}**
Os1	*6h*	6682(1)	9975(1)	2500	3.9(1)
Bi1	*6g*	6712(1)	10000	5000	4.5(1)
Bi2	*4f*	3333	6667	6051(1)	7.4(1)
Na1	*2b*	10000	10000	2500	17(1)
Na2	*12i*	2976(6)	9912(4)	3789(2)	13(1)
Na3	*4f*	3333	6667	3731(4)	16(1)
O1	*6h*	8035(11)	12028(12)	2500	10(1)
O2	*6h*	5648(8)	7895(11)	2500	10(1)
O3	*12i*	5336(5)	7486(6)	5037(3)	7.3(8)
O4	*12i*	5443(6)	10127(6)	3631(4)	8.1(9)
O5	*12i*	7987(7)	9862(7)	3703(4)	8.3(9)

Table S6. Selected bond distances for $Na_9Bi_5Os_3O_{24}$.

**Atom pairs**	***Distance* (Å)**	**Atom pairs**	***Distance* (Å)**
Os1-O2 (×1)	1.767(10)	Na1-O5 (×6)	2.457(5)
Os1-O1 (×1)	1.773(11)	Na2-O4 (×1)	2.335(7)
Os1-O4 (×2)	1.944(5)	Na2-O1 (×1)	2.406(9)
Os1-O5 (×2)	2.044(5)	Na2-O3 (×1)	2.440(6)
Bi1-O5 (×2)	2.129(5)	Na2-O5 (×1)	2.441(7)
Bi1-O3 (×2)	2.140(5)	Na2-O2 (×1)	2.480(8)
Bi1-O4 (×2)	2.191(5)	Na2-O3 (×1)	2.490(6)
Bi2-O3 (×3)	2.150(5)	Na3-O2 (×3)	2.396(6)
Bi2-O1 (×3)	2.937(5)	Na3-O3 (×3)	2.525(6)

Table S7. Anisotropic displacement parameters ($Å^2$ x 10^3) for $Na_9Bi_5Os_3O_{24}$, ordered structure model. The anisotropic displacement factor exponent takes the form: $-2\pi^2[h^2a^{*2}U_{11} + ... + 2hka^*b^*U_{12}]$.

**Atom**	**U11**	**U22**	**U33**	**U23**	**U13**	**U12**
Os	5(1)	5(1)	2(1)	0	0	2(1)
Bi1	6(1)	5(1)	2(1)	0(1)	0(1)	2(1)
Bi2	9(1)	9(1)	5(1)	0	0	4(1)
Na1	15(2)	15(2)	20(3)	0	0	7(1)
Na2	9(1)	14(2)	12(2)	1(1)	0(1)	3(1)
Na3	3(1)	3(1)	43(4)	0	0	1(1)
O1	14(4)	3(3)	9(2)	0	0	2(2)
O2	15(3)	2(3)	9(2)	0	0	1(3)
O3	9(2)	5(2)	8(2)	1(2)	2(2)	4(2)
O4	6(2)	14(2)	6(2)	-1(2)	1(2)	6(2)
O5	9(2)	14(2)	4(2)	1(2)	0(2)	7(2)

Table S8. Crystal data and structure refinement for $Na_9Bi^{V}_3Bi^{III}_2Os_3O_{24}$ assuming disorder.

**Crystal**	**1**	**2**
Identification code	HR2212_0m	nus313a_0m
Empirical formula	$Bi_5Na_9O_{24}Os_3$	
Formula weight [g/mol]	2206.41	
T [K]	296(2)	
Crystal data		
Crystal system, space group	Hexagonal, $P\bar{6}2c$	
a [Å]	9.8115(3)	9.8344(2)
c [Å]	12.8457(5)	12.8732(5)
V [$Å^3$]	1070.93(8)	1078.23(6)
Z, d_{calc} [g/cm^3]	2, 6.842	2, 6.796
$\mu(MoK_a)$ [mm^{-1}]	58,944	58,545
F(000)	1868	
Data collection		
$2\Theta_{max}$	70°	
Reflections collected	75234	18635
Reflections unique, R_{int}	1637, 0.0877	1639, 0.0508
Refinement		
Data / restraints / parameters	1637 / 0 / 75	1639 / 0 / 76
Goodness-of-fit on F^2	1,037	1,055
R_1, wR2 [I > 2s(I)]	0.0176, 0.0421	0.0242, 0.0911
R_1, wR2 [all data]	0.0234, 0.0451	0.0288, 0.0965
Absolute structure parameter	-0.033(11)	0.024(11)
Extinction coefficient	0.00144(6)	0.00076(10)
±D [$eÅ^{-3}$]	1.728/-1.905	2.687/-3.289
Structure model		
Na/Bi-disorder	yes	yes
Degree of anti-site disorder	96.53%/3.47%	39.58%/60.42%

Table S9. Atomic coordinates (x 10^4) and equivalent isotropic displacement parameters (A^2 x 10^3) for crystal 1 and 2 of $Na_9Bi_5Os_3O_{24}$ obtained after refinement (top rows) and with anti-site disorder (bottom rows). U(eq) is defined as one third of the trace of the orthogonalized Uij tensor.

**Atom**	**Sample**	**Bi/Na-disorder**	**Wyckoff**	**Occ.**	***x***	***y***	***z***	**U(eq)**
Os	1	yes	*6h*		6682(1)	9975(1)	2500	5(1)
	2	yes			3300(1)	-8(1)	2500	9(1)
Bi1	1	yes	*6g*		6711(1)	10000	5000	5(1)
	2	yes			3294(1)	0	5000	10(1)
Bi2A	1	yes	*4f*	0.965(3)	3333	6667	6051(1)	7(1)
	2	yes		0.396(3)	6667	3333	3961(1)	16(1)
Bi2B	1	yes	*4f*	0.035	3333	6667	3997(11)	11(3)
	2	yes		0.604	6667	3333	6043(1)	10(1)
Na1	1	yes	*2b*		10000	10000	2500	19(1)
	2	yes			0	0	7500	28(2)
Na2	1	yes	*12i*		2994(5)	9916(3)	3788(2)	13(1)
	2	yes			6949(8)	-24(4)	6211(3)	20(1)
Na3A	1	yes	*4f*	0.965(3)	3333	6667	3654(6)	19(2)
	2	yes		0.396(3)	6667	3333	6370(20)	57(13)
Na3B	1	yes	*4f*	0.035	3333	6667	6440(140)	19(2)
	2	yes		0.604	6667	3333	3581(15)	47(6)
O1	1	yes	*6h*		8032(12)	12026(11)	2500	12(1)
	2	yes			2159(18)	-2079(15)	7500	22(2)
O2	1	yes	*6h*		5649(7)	7892(10)	2500	12(1)
	2	yes			4127(11)	2040(14)	7500	22(2)
O3	1	yes	*12i*		5337(5)	7490(5)	5037(3)	9(1)
	2	yes			4649(6)	2510(6	5010(4)	11(1)
O4	1	yes	*12i*		5443(5)	10119(5)	3632(3)	10(1)
	2	yes			4624(7)	32(8)	6365(5)	18(1)
O5	1	yes	*12i*		7983(6)	9859(6)	3703(3)	10(1)
	2	yes			1925(8)	-36(8)	6290(4)	17(1)

Table S10. Summary of bond lengths [Å] obtained from single crystal structure refinement using different protocols of refinement for all crystal samples

**Crystal**	**1**	**2**
**Na/Bi-disorder**	yes	yes
**Degree of disorder**	96.53%/3.47%	39.58%/60.42
Os1-O2 (×1)	1.770(9)0	1.755(13)
Os1-O1 (×1)	1.771(10)	1.767(14)
Os1-O4 (×2)	1.945(4)	1.944(6)
Os1-O5 (×2)	2.043(4)	2.054(6)
Bi1-O5 (×2)	2.128(4)	2.126(6)
Bi1-O3 (×2)	2.136(4)	2.140(5)
Bi1-O4 (×2)	2.190(4)	2.182(6)
Bi2A-O3 (×3)	2.151(4)	2.193(5)
Bi2B-O3 (×3)	2.172(10)	2.180(5)
Na1-O5 (×6)	2.459(4)	2.466(7)
Na2-O4 (×1)	2.319(6)	2.323(10)
Na2-O1 (×1)	2.404(8)	2.465(12)
Na2-O3 (×1)	2.440(5)	2.463(7)
Na2-O5 (×1)	2.453(6)	2.580(9)
Na2-O2 (×1)	2.478(7)	2.447(12)
Na2-O3 (×1)	2.487(5)	2.450(6)
Na2-O5 (×1)		2.667(9)
Na3A-O2 (×3)	2.464(6)	2.608(18)
Na3A-O3 (×3)	2.467(7)	2.46(2)
Na3B-O3 (×3)	2.49(13)	2.524(15)
Na3B-O1 (×3)	2.65(9)	2.493(13)
	= affected by disorder	

Table S11. Anisotropic displacement parameters (Å^2 × 10^3) for $Na_9Bi_5Os_3O_{24}$ assuming anti-site disorder (crystal 1). The anisotropic displacement factor exponent takes the form: $-2\pi^2[h^2a^{*2} U_{11} + ... + 2hka^*b^*U_{12}]$.

Atom	U11	U22	U33	U23	U13	U12
Os1	6(1)	5(1)	3(1)	0	0	3(1)
Bi1	7(1)	6(1)	3(1)	0(1)	0(1)	3(1)
Bi2A	9(1)	9(1)	5(1)	0	0	4(1)
Bi2B	12(3)	12(3)	8(7)	0	0	6(2)
Na1	17(2)	17(2)	23(3)	0	0	9(1)
Na2	12(1)	12(2)	12(2)	0(1)	-1(1)	4(1)
Na3A	19(2)	19(2)	19(3)	0	0	10(1)
Na3B	19(2)	19(2)	19(3)	0	0	10(1)
O1	15(4)	6(3)	10(2)	0	0	3(2)
O2	17(3)	5(3)	10(2)	0	0	3(3)
O3	10(2)	7(2)	10(2)	1(2)	3(1)	4(2)
4O4	8(2)	15(2)	8(2)	-1(2)	0(1)	7(2)
O5	11(2)	16(2)	5(2)	1(2)	1(1)	8(2)

Table S12. Anisotropic displacement parameters (Å^2 × 10^3) for $Na_9Bi_5Os_3O_{24}$ assuming inversion and disorder (crystal 2). The anisotropic displacement factor exponent takes the form: $-2\pi^2[h^2a^{*2} U_{11} + ... + 2hka^*b^*U_{12}]$.

Atom	U11	U22	U33	U23	U13	U12
Os1	10(1)	10(1)	8(1)	0	0	5(1)
Bi1	12(1)	10(1)	8(1)	0(1)	0(1)	5(1)
Bi2A	16(1)	16(1)	16(1)	0	0	8(1)
Bi2B	12(1)	12(1)	9 (1)	0	0	6(1)
Na1	21(2)	21(2)	43(5)	0	0	10(1)
Na2	21(2)	16(2)	22(2)	1(1)	0(1)	8(2)
Na3A	80(19)	80(19)	13(10)	0	0	40(10)
Na3B	62(9)	62(9)	15(6)	0	0	31(4)
O1	30(6)	11(4)	15(3)	0	0	3(3)
O2	37(4)	5(4)	16(3)	0	0	5(4)
O3	15(2)	5(2)	14(2)	0(2)	0(1)	5(2)
O4	15(3)	26(3)	15(2)	-1(2)	0(2)	13(2)
O5	18(3)	27(3)	9(2)	1(2)	1(2)	14(3)

Table S13. DFT+U optimized crystal structure (unit cell volume and shape, atomic positions were allowed to relax, number of electrons decreased by 2 per each Os (compensated by external charges). Space group: $P\bar{6}2c$, a = 9.01577 Å, c = 12.38993 Å.

**Atom**	**Wyckoff**	***x***	***y***	***z***
Os1	*6h*	0.66543	-0.02727	0.2500
Bi1	*6g*	0.66530)	0.0000	0.000
Bi2	*4f*	0.3333	0.6667	0.62392
Na1	*2b*	0.0000	0.0000	0.2500
Na2	*12i*	0.28790	-0.0023	0.37657
Na3	*4f*	0.3333	0.6667	0.37511
O1	*6h*	0.77653	0.24262	0.2500
O2	*6h*	0.54505	0.75121	0.2500
O3	*12i*	0.53331	0.73165	0.50826
O4	*12i*	0.52839	0.00057	0.35882
O5	*12I*	0.81560	0.00601	0.35957

3. Figures

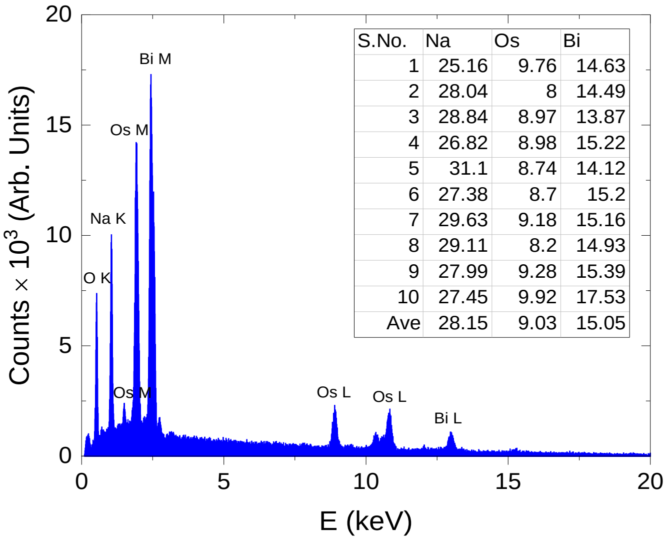

S.No.	Na	Os	Bi
1	25.16	9.76	14.63
2	28.04	8	14.49
3	28.84	8.97	13.87
4	26.82	8.98	15.22
5	31.1	8.74	14.12
6	27.38	8.7	15.2
7	29.63	9.18	15.16
8	29.11	8.2	14.93
9	27.99	9.28	15.39
10	27.45	9.92	17.53
Ave	28.15	9.03	15.05

Figure S1: Elemental analysis of a typical crystal of $Na_9Bi_5Os_3O_{24}$ using SEM-EDX. Inset shows table of atomic ratios at different regions.

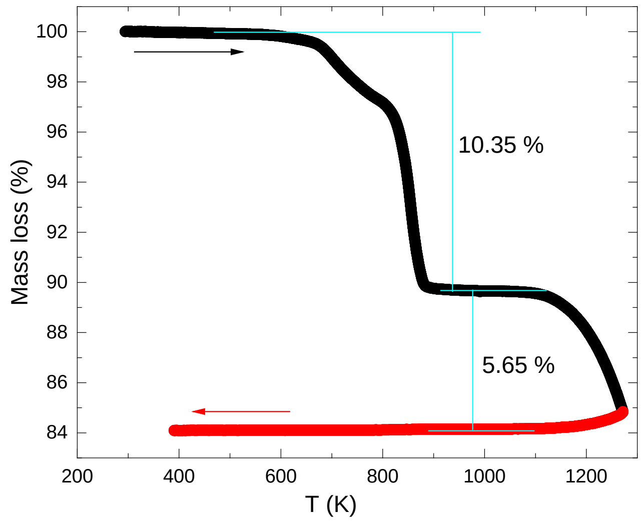

Figure S2. Thermal decomposition profile of $Na_9Bi_5Os_3O_{24}$.

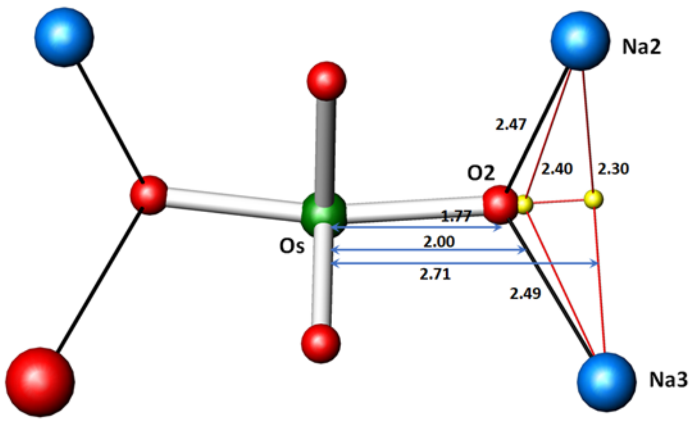

Figure S3. Demonstrating lack of structural frustration with respect to short apical Os-O2 bond. The distances are in Å.

4. References

[1] TOPAS Version 6, Bruker AXS, Karlsruhe, Germany, **2017**.

[2] G. Oszlányi, A.Süto, *Acta Cryst.* **2004**, *A60*, 134-141.

[3] J. Karle, H. Hauptman, *Acta Cryst*. **1956**, *9*, 635-651.

[4] Bruker Suite, version 2013/1. Bruker AXS Inc., Madison, WI, **2013**.

[5] SADABS — Bruker AXS area detector scaling and absorption, version 2016/2, Krause L.; Herbst-Irmer, R.; Sheldrick, G. M. *J. Appl. Cryst*. **2015**, *48*, 3-10.

[6] G. M. Sheldrick, *Acta Crystallogr., Sect. A: Found. Crystallogr.* **2008**, *64*, 112-122.

[7] G. M. Sheldrick, *Acta Crystallogr., Sect. C: Struct. Chem.* **2015**, *71*, 3-8.

[8] J. P. Perdew, K. Burke, M Ernzerhof, *Phys. Rev. Lett*. **1996**, *77*, 3865.

[9] D. F. Mosca, L. V. Pourovskii, B. H. Kim, P. Liu,; S. Sanna, F. Boscherini, S. Khmelevskyi, C. Franchini, Accepted, *Phys. Rev. B*, **2021**, http://arxiv.org/abs/2102.10839.

[10] O. K Andersen, O. Jepsen, *Phys. Rev. Lett*. **1984**, *53*, 2571.
